# Case Report: Tuberculous abscess of the popliteal fossa: A case report

**DOI:** 10.12688/f1000research.138152.1

**Published:** 2023-09-25

**Authors:** Hedi Belhassen, Mohamed Ali Khlif, Mohamed Achraf Ferjani, Yosri Abcha, Maher Barsaoui

**Affiliations:** 1 Orthopedic department, La Rabta Hospital, Tunis, 1007, Tunisia

**Keywords:** Tuberculosis, knee, abscess, diagnosis, therapeutic

## Abstract

Introduction

Tuberculosis of the soft tissues is a rare form of extra pulmonary tuberculosis, and isolated localization in the popliteal fossa is particularly exceptional. Atypical clinical presentation can lead to delayed diagnosis and serious complications.

Case report

We report the case of a 17-year-old patient who was diagnosed with tuberculosis of the popliteal fossa. He presented with a painful inflammatory swelling of the right popliteal fossa associated with a homolateral inguinal lymph node, without knee joint effusion. Standard chest and knee X-rays were normal, while MRI showed an 8 cm well vascularized, partly liquefied mass in the popliteal fossa, developed in contact with the semimembranosus and medial gastrocnemius muscles, associated with a popliteal lymph node without synovial effusion or thickening. Microbiological tests did not isolate any germs. The diagnosis was made on histological examination after biopsy, which revealed a caseous granuloma surrounded by epithelioid cells. The patient was treated with anti-tuberculosis therapy for 9 months. The clinical and radiological regression of the swelling was observed without recurrence at 2 years of follow-up.

Conclusion

Any soft tissue abscess should raise suspicion of tuberculosis, especially in endemic countries. The importance of histopathological examination should be emphasized to establish the diagnosis in the absence of signs in favor of a primary localization.

## Introduction

Osteoarticular tuberculosis remains prevalent in countries where tuberculosis is endemic. It represents 1 to 5% of all forms of the disease. It predominantly affects the spine (40%), hip (25%), and knee (8%). ^1^^,^^2^

The symptoms of the extra-spinal form are non-specific, with an insidious clinical picture and inconsistent general signs, which explains the diagnostic difficulties. Early management is necessary to prevent serious complications. ^3^ In the knee, tuberculosis typically presents as joint involvement: synovitis, arthritis, or an infected Baker’s cyst, and rarely as extra-articular involvement. ^4^^,^^5^

Currently, antitubercular antibiotics are the standard treatment. Moreover, surgical treatment is limited to cases resistant to medical treatment. ^4^

## Case report

This is a 17-year-old male, Tunisian high school student, from central-western Tunisia (Sbeïtla, Kasserine) with no notable medical history but with a history of exposure to tuberculosis seven years ago (his brother had pulmonary tuberculosis) and a history of regular consumption of unpasteurized milk. The patient presented with a painful, red, hot swelling in the right popliteal fossa that was resistant to first-line medical treatment with analgesics. Furthermore, he reported a weight loss of 5 kilograms over the past 2 months, as well as fever and night sweats over the past 2 weeks.

On examination, the patient had a limp due to an antalgic stance in the knee flessum. An inflammatory swelling of 10 cm in diameter was found in the right popliteal fossa. It was a painful, soft, and mobile swelling. In addition, there was no joint effusion in the knee and no metaphyseal pain in the femur and tibia. An inguinal lymph node on the same side was found.

**Figure 1.**
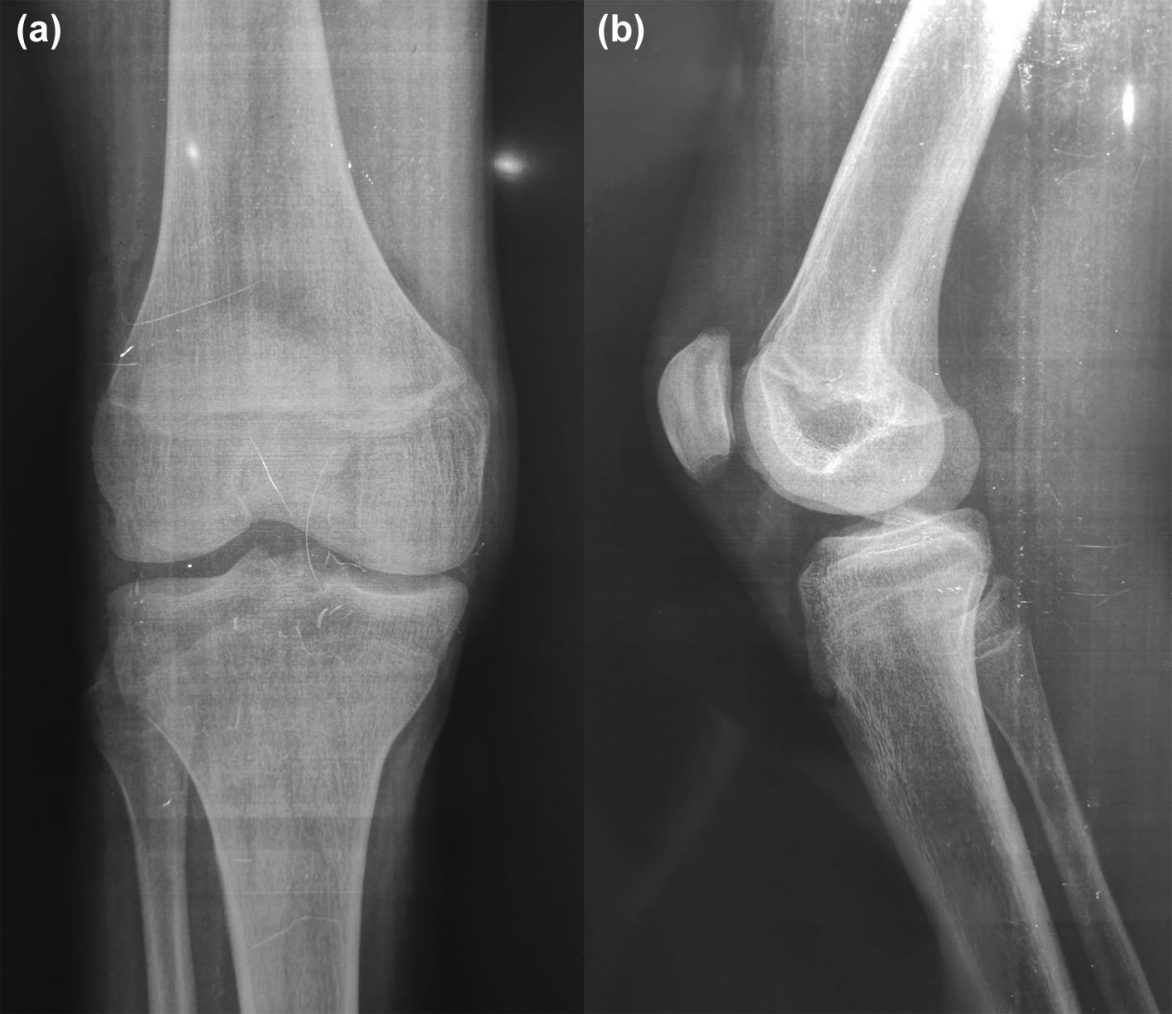
A radiograph of the right knee of a 17-year-old boy showing no detectable abnormalities.

**Figure 2.**
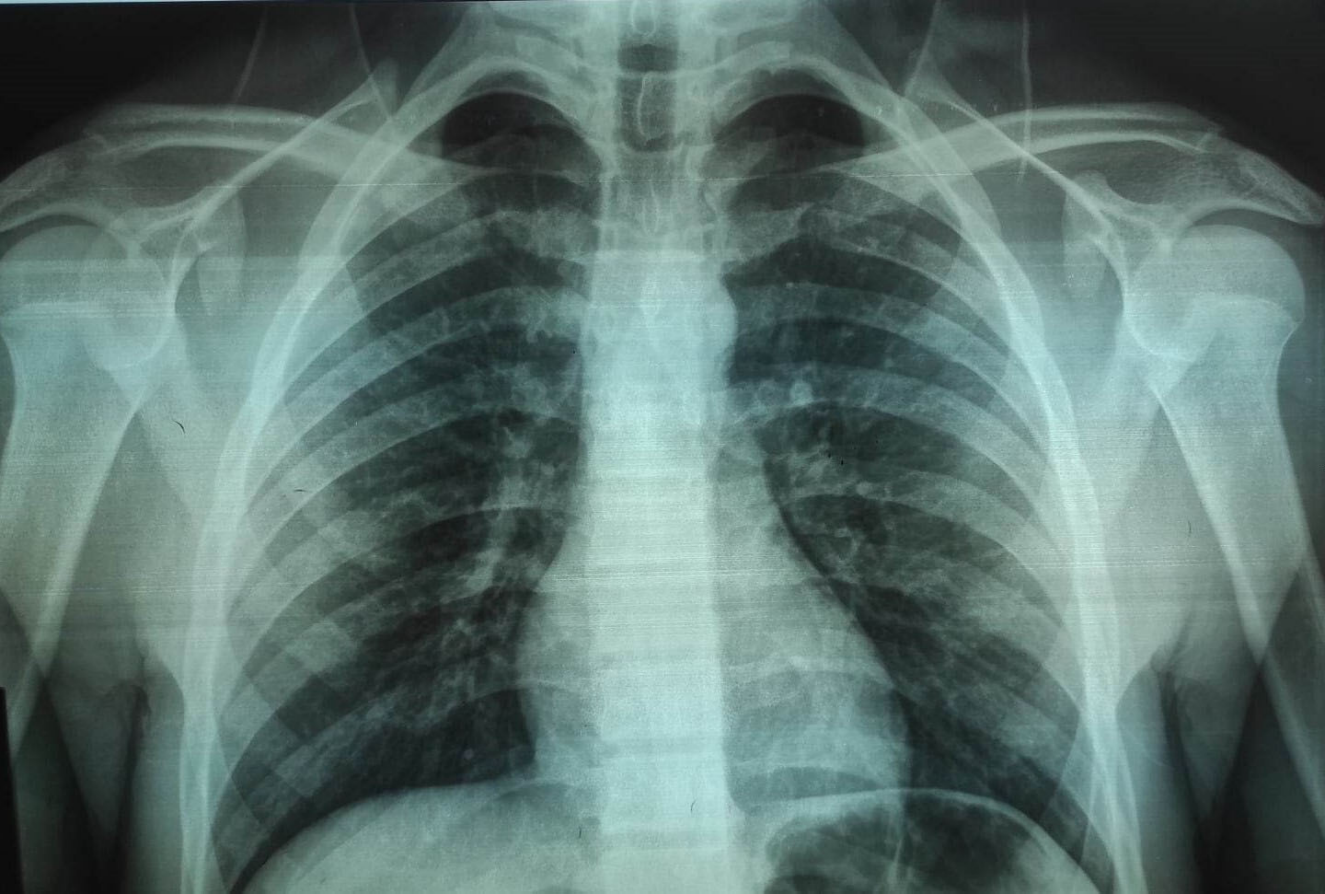
A chest radiograph of a 17-year-old boy showing no detectable abnormalities.

Laboratory tests showed a biological inflammatory syndrome with a white blood cell count of 12,040 and a C-reactive protein level of 67.3 mg/L. Chest and knee radiographs were normal ( Figures 1 and 2).

**Figure 3.**
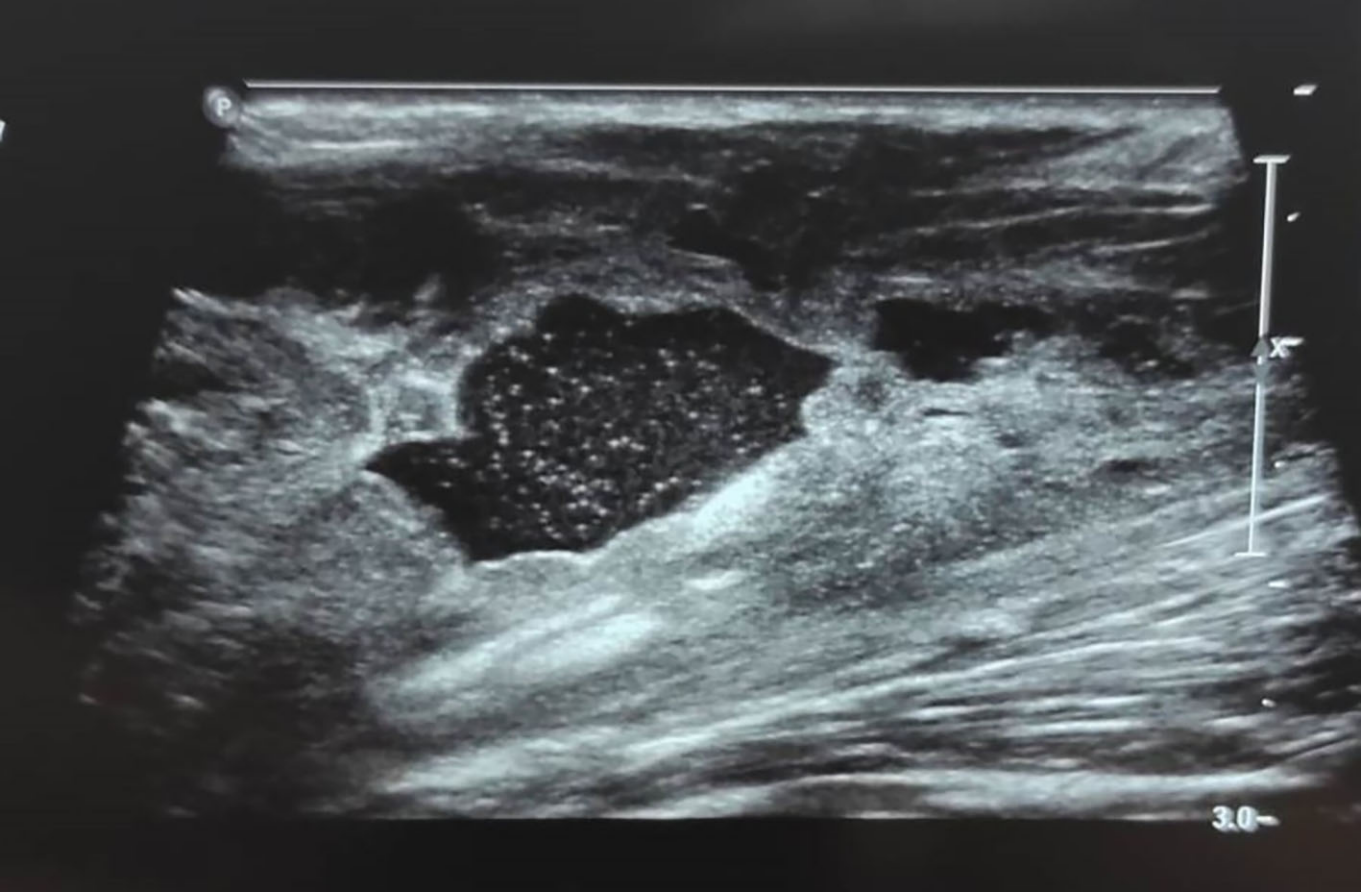
Utrasonography of the right knee reveals an echogenic collection of the right popliteal fossa.

Doppler ultrasound of the lower limb showed a collection of the right popliteal fossa with an echogenic content, a clean wall independent of the popliteal pedicle, and thrombophlebitis of the right saphenous vein ( Figure 3). The patient was treated with anticoagulant therapy in the form of low-molecular-weight heparin overlap and Sintrom (acenocumarol).

**Figure 4.**
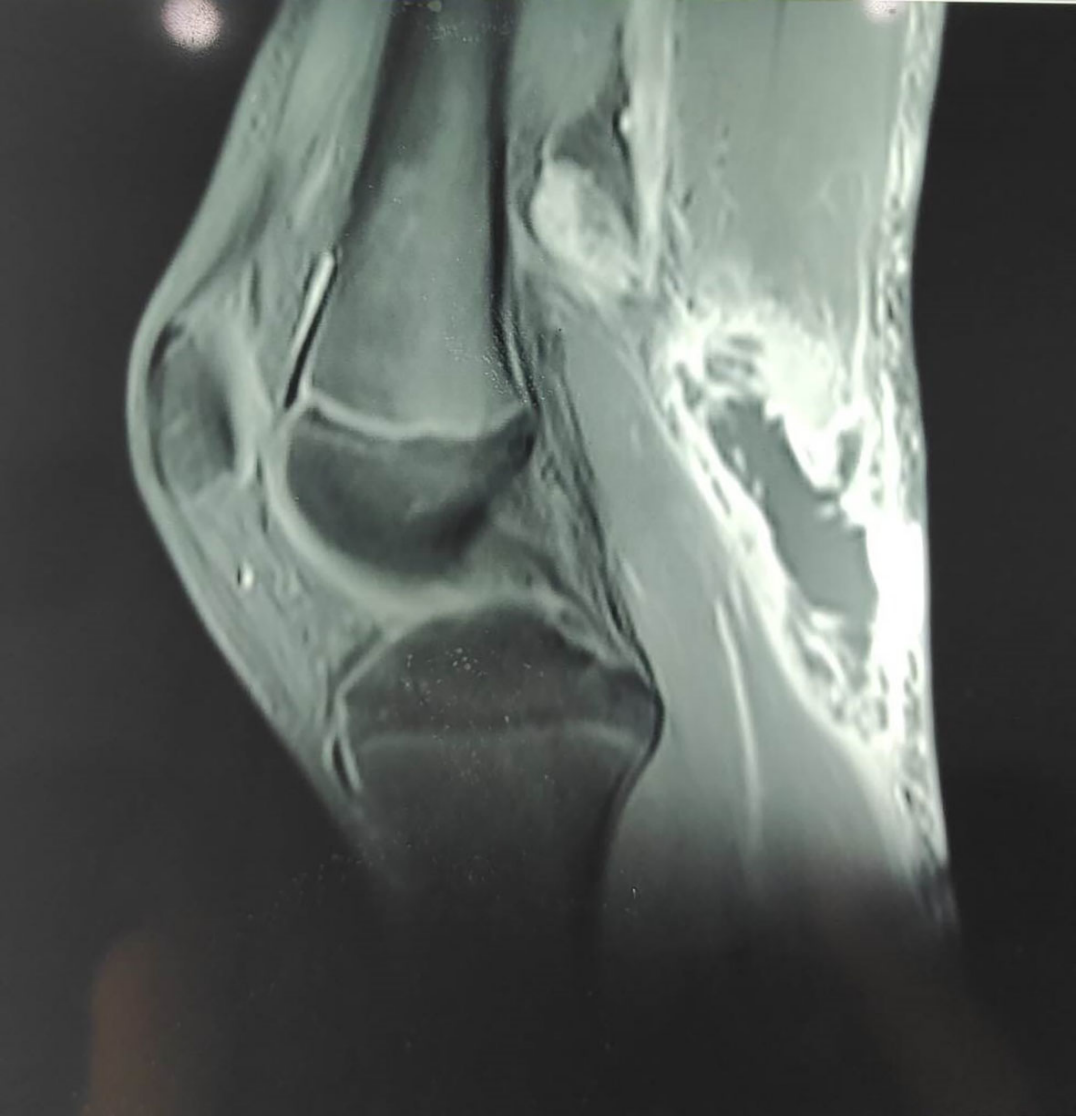
T1-weighted MRI reveals a vascularized mass adjacent to the semimembranosus and medial gastrocnemius muscles.

A magnetic resonance image (MRI) showed a well-vascularized mass of 8 cm in diameter that was partly liquefied and developed in contact with the semimembranosus and medial gastrocnemius muscles, popliteal vessels, and posterior tibial nerve, with muscle and adipose edema and popliteal lymphadenopathy but no synovial effusion or thickening. This aspect suggested an infectious etiology ( Figure 4).

The etiological investigation showed negative tuberculin skin test, negative direct examination and culture for Koch’s bacillus in urine and sputum, negative Rose Bengal serology and negative Wright serology.

**Figure 5.**
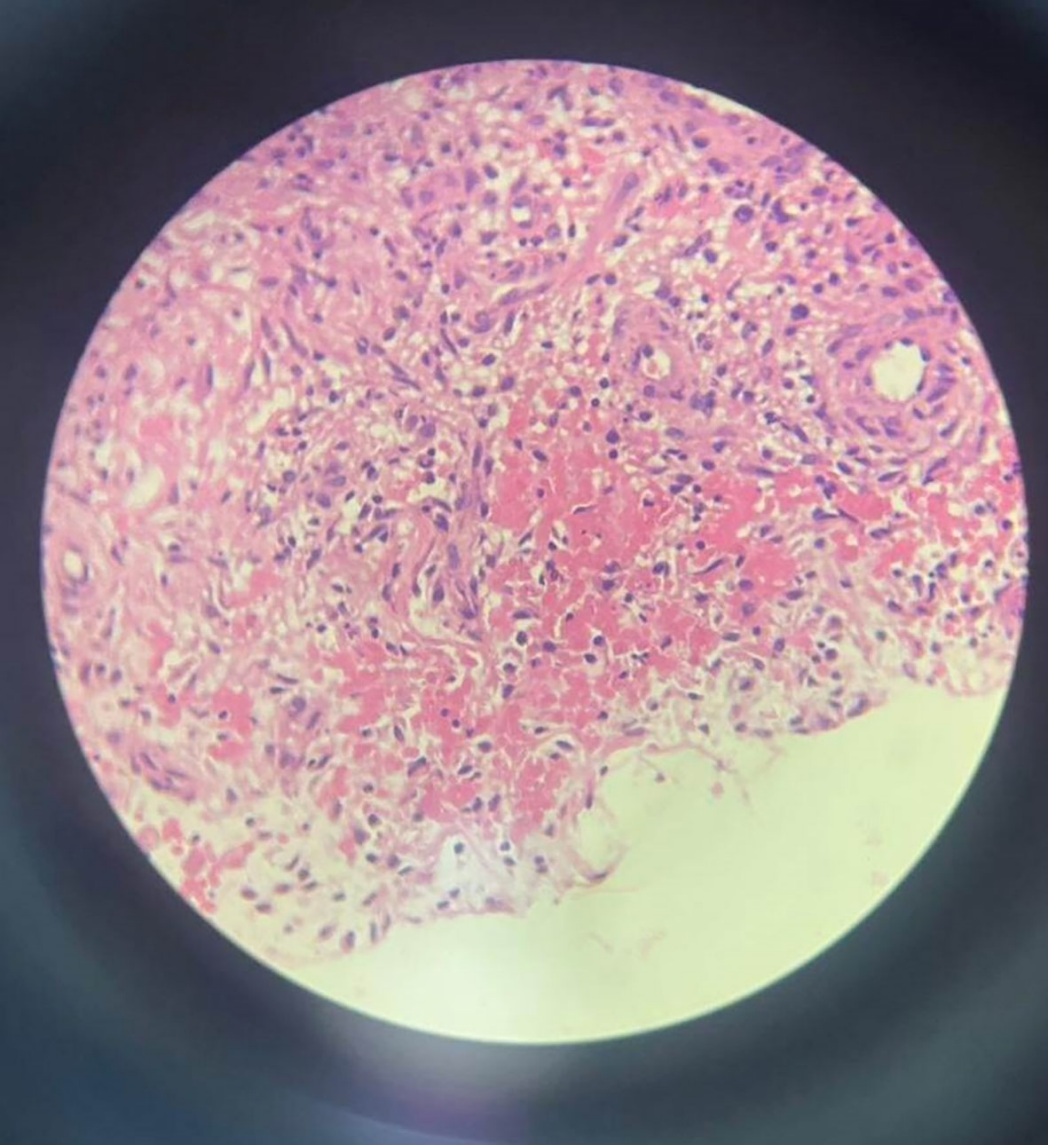
Microscopy shows an inflammatory granulation tissue with acellular eosinophil deposits and epithelioid cells.

An ultrasound-guided biopsy puncture was done. The bacteriological results were negative. Histopathological examination revealed the presence of inflammatory granulation tissue with acellular eosinophil deposits and epithelioid cells, suggesting a tuberculous origin ( Figure 5).

**Figure 6.**
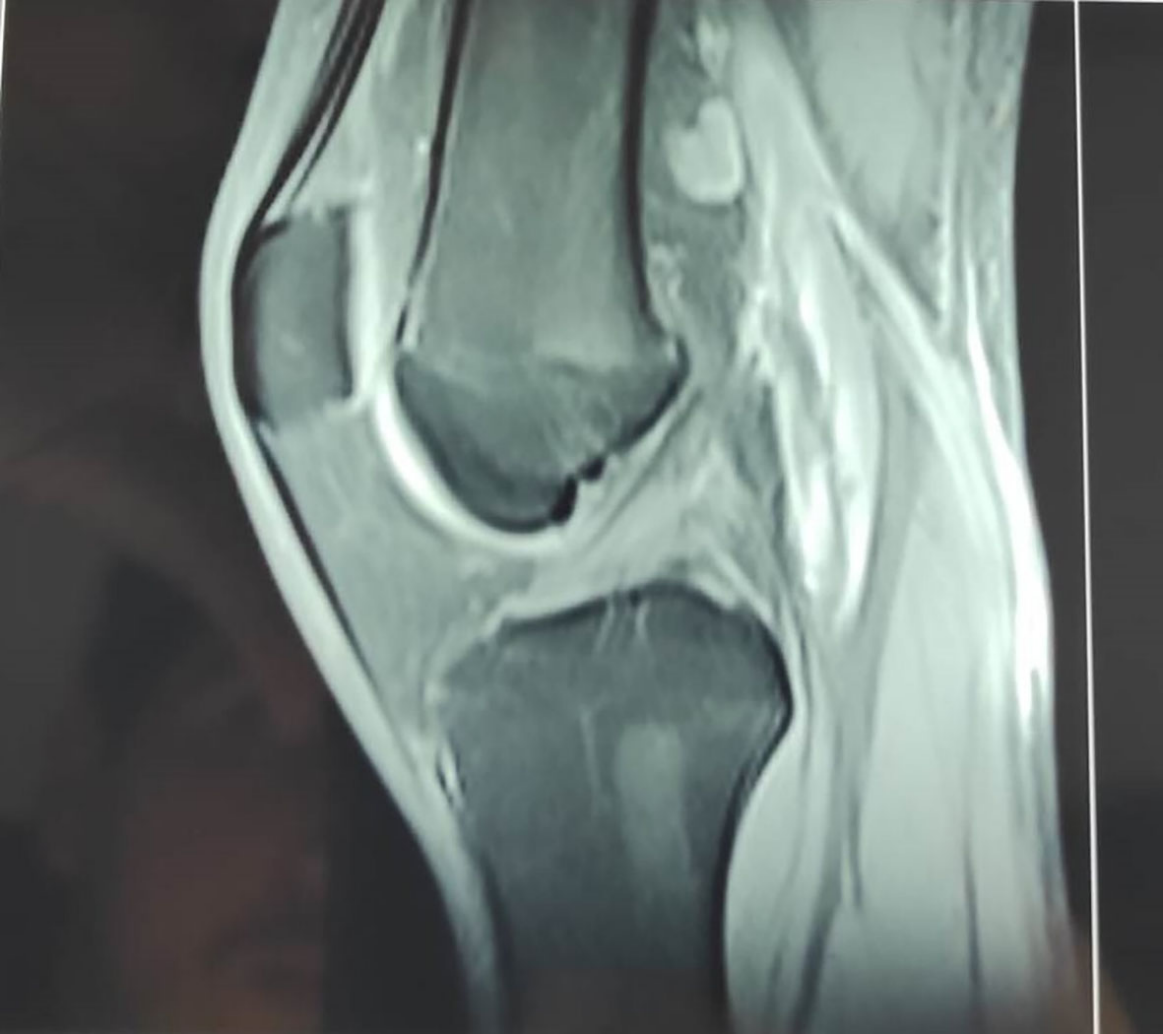
A control MRI demonstrated complete disappearance of the previously described mass with no joint effusion.

The decision was to start an antituberculosis trial treatment with isoniazid (H), rifampicin (R), pyrazinamide (Z), ethambutol (E) and a pre-treatment assessment. The patient was treated with anti-tubercular drugs for 9 months according to the 2HRZE/7HR regimen with regular monitoring of liver and hematological function. The dosage was 4 HRZE tablets per day associated with 3/4 Sintrom tablets to control the thrombophlebitis of the internal saphenous vein. The patient’s condition improved, and a control MRI was performed in the eighth week of treatment that demonstrated complete disappearance of the previously described mass in the popliteal fossa, with no joint effusion or synovial thickening. Muscular and fatty edema had disappeared, and the bone signal was normal ( Figure 6).

**Figure 7.**
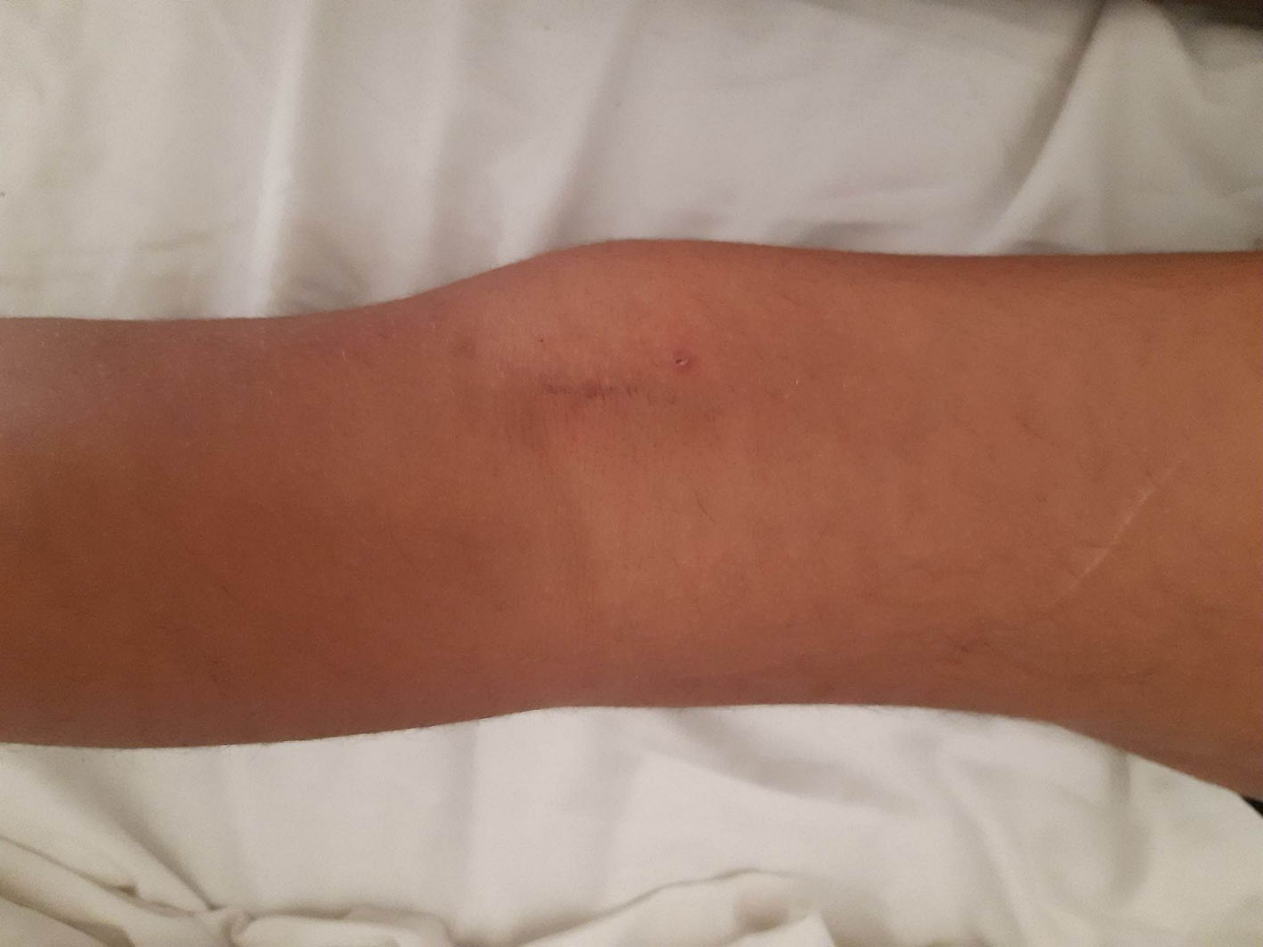
A clinical photograph of the knee showing a posterior scar with disappearance of the swelling.

With 2 years of follow-up, the clinical and radiological evolution was favorable ( Figure 7): clinical examination showed complete disappearance of the swelling and inguinal lymphadenopathy, with no joint effusion and full active mobility of the knee, and radiography revealed no bone lesions.

## Discussion

Soft tissue tuberculosis, defined as involvement of tendons, bursae, muscles, or deep fascia, is a rare form of musculoskeletal tuberculosis. It is usually associated with immunocompromised patients. ^6^ Isolated localization in the popliteal fossa is also exceptional. ^7^

Several authors ^8^^–^^10^ have reported in the literature that isolated or primary soft tissue tuberculosis without bone involvement in immunocompetent patients represents a rare form of extrapulmonary tuberculosis and its exact incidence is not known. Its clinical manifestations can mimic malignant diseases or other inflammatory diseases, making diagnosis difficult. The main differential diagnoses are pyogenic abscesses, atypical mycobacterial abscesses, hydatid cysts, and certain tumors. ^11^

It is interesting to note that most cases of soft tissue tuberculosis reported in the literature were located near joints and bursae. The pathophysiology of this isolated involvement remains difficult to understand. ^12^ However, extension from an adjacent joint, bone, or bursa and even direct inoculation have all been reported. In this context, Lupatkin et al. ^13^ reported that tuberculous abscesses resulted from hematogenous, lymphatic, or local contamination from adjacent or other primary infection areas and, in rare cases, direct inoculation.

In our case, there was no clear explanation for the focal origin of the popliteal fossa involvement. Furthermore, this isolated and primary involvement could be attributed to hematogenous spread or extension from neighboring bursae. This hypothesis is most likely due to the presence of nearly 13 bursae around the knee.

Tuberculosis of soft tissues mainly affects the extremities, presenting as localized masses accompanied by local inflammatory signs and pain. General symptoms such as fever and weight loss are inconsistent. ^14^^,^^15^ Recent cases of soft tissue tuberculosis published in the last 10 years were identified by conducting a search on PubMed ( Table 1). We found 17 reported cases in the literature, including 10 males and seven females. The patients ranged from 7 to 79 years old. 12 cases were reported in Asian countries, four cases in African countries, and one case in America. In 13 patients, the lesions were predominantly in the extremities including the thigh, calf, forearm, and wrist. The other four patients had lesions in the buttocks, back, chest, and iliopsoas. The diagnosis of tuberculosis was made by a combination of bacteriological and pathological examination. The majority of cases improved under medical treatment based on antituberculosis drugs without any associated surgical procedure.

**Table 1.** Main characteristics of reported soft tissue tuberculosis cases. PCR: Polymerase chain reaction, NS: Not specified.

Reference	Year and country	Age and gender	Predisposing factors	Location	Clinical signs	Diagnosis	Treatment
Arora *et al.* ^16^	2012 India	15/male	NS	Left thigh	Swelling + systemic signs	Bacteriological examination	Medical treatment for 6 months
Lee *et al.* ^17^	2013 Korea	62/male	NS	Right thigh	Swelling	Bacteriological and histological examination	Medical treatment for 6 months
Elshafie *et al.* ^18^	2013 Oman	25/male	History of tuberculosis exposure	Right buttock	Swelling	Bacteriological and histological examination	Drainage followed by 9-month antibiotic
Neogi *et al.* ^19^	2013 India	11/female	NS	Right thigh and calf/Left arm	Swelling	Bacteriological and histological examination	Medical treatment for 6 months
Meena *et al.* ^20^	2015 India	25/male	NS	Right arm	Swelling	BK PCR	Medical treatment
Dhakal *et al.* ^21^	2015 Nepal	9/female	NS	Right forearm and calf	Swelling	Bacteriological and histological examination	Medical treatment
Sbai *et al.* ^22^	2016 Tunisia	45/male	NS	Right wrist	Swelling	Bacteriological and histological examination	Drainage followed by 8-month antibiotic
Al-khazraji *et al.* ^23^	2017 USA	33/female	Lupus nephritis	Right calf	Inflammatory swelling	Bacteriological and histological examination	Drainage followed by antibiotics
Alaya *et al.* ^24^	2017 Tunisia	23/female	History of tuberculosis exposure	Right thigh	Inflammatory swelling + pain	BK PCR + Histological examination	Medical treatment for 12 months
Manicketh *et al.* ^14^	2018 India	55/female	Pulmonary tuberculosis	Left wrist and right calf	Inflammatory swelling with systemic signs	Bacteriological examination	Medical treatment
Hashimoto *et al.* ^25^	2018 Japan	79/male	NS	Left wrist	Inflammatory swelling	Histological examination	Drainage followed by antibiotics
Zeng *et al.* ^26^	2019 China	49/male	Pulmonary tuberculosis, corticosteroid therapy	Both thighs and calves	Inflammatory swelling	BK PCR + Bacteriological and histological examination	Medical treatment
Zitouna *et al.* ^27^	2019 Tunisia	42/female	NS	Right lumbar paraspinal muscles	Swelling	Bacteriological and histological examination	Medical treatment
Moyano *et al.* ^28^	2019 Senegal	29/male	NS	Right hemithorax	Increased volume of the hemithorax	Bacteriological examination + BK PCR	Medical treatment
Fahad *et al.* ^29^	2020 Pakistan	45/Female	NS	Right forearm	Swelling	Histological examination	
Murugesh *et al.* ^30^	2020 India	31/male	Renal transplantation under immunosuppressants	Right calf and foot	Inflammatory swelling	Bacteriological and histological examination	Drainage followed by antibiotics
Tone *et al.* ^15^	2021 Japan	29/male	Tuberculous pleurisy	Right iliopsoas muscle	Pain and fever	Bacteriologial and histological examination + BK PCR	Percutaneous drainage followed by antibiotics

In the literature, ^7^ to confirm a definitive diagnosis, it is essential to identify the Koch bacillus. However, tuberculous soft tissue disease is paucibacillary. Often, the Ziehl-Nielsen test is negative and it is necessary to wait for the results of Lowenstein culture. Histological examination is more sensitive than microbiological tests and can confirm the diagnosis in 80% of cases. ^31^ A normal chest radiograph, the absence of systemic symptoms, or the absence of detectable tuberculosis focus should not deter from considering this diagnosis.

In our case, faced with a history of tuberculosis exposure and a painful isolated swelling of the popliteal fossa, we performed an ultrasound-guided biopsy puncture of the swelling. The diagnosis was finally established by histopathology, which showed pathognomonic caseating granulomas of tuberculosis.

In the literature, ^10^ the treatment protocol is not clearly defined. The main debated questions concern the duration of medical treatment as well as the need and modalities of any surgical treatment. Some authors ^32^^,^^33^ advocated for primary medical treatment with a combination of antituberculous antibiotics. The choice of drugs is generally the same as for pulmonary tuberculosis (isoniazid, rifampicin, pyrazinamide, and ethambutol). This treatment is divided into two phases: an initial phase based on quadritherapy that lasts two months and a secondary phase with a bitherapy that lasts 4 to 7 months or longer in extensive cases. Moreover, surgical drainage is reserved for cases resistant to medical treatment. ^16^ Postoperative outcomes are marked by local recurrence in 50% of cases within a year. ^25^ Indeed, our patient responded well to exclusive medical treatment for 9 months. The evolution was spectacular with complete regression of the mass and no recurrence with a follow-up of 2 years. The patient expressed a high level of satisfaction regarding the clinical outcomes following the treatment. He reported a successful recovery and the ability to resume his normal physical activity level.

Our case study had several strengths: we conducted a meticulous clinical examination, along with radiological investigations using ultrasound and MRI of the knee. Given that we are in an endemic country and taking into account the patient’s history of tuberculosis exposure, the diagnosis of tuberculous abscess should always be considered as a first possibility. Therefore, we decided to perform a biopsy to confirm the etiology before proceeding with surgery. After confirming the diagnosis through histology and reviewing the literature, we opted for antibiotic treatment and monitoring of the progression. Consequently, the lesion regressed totally overtime.

## Conclusion

Tuberculosis of soft tissues is a rare form of the disease. It is even rarer for it to present as a primary abscess in the popliteal fossa. The lack of specificity of clinical and radiological signs and the insidious and progressive course make the diagnosis difficult.

This case report emphasizes the importance of considering the diagnosis of tuberculosis in the differential diagnosis of any unexplained soft tissue swelling in endemic areas, despite the absence of systemic and pulmonary symptoms. Thus, a more thorough investigation can prevent delayed diagnosis and its devastating complications.

## Consent

Written informed consent for the publication of the patient’s clinical details and clinical images was obtained from the patient and his parents.

## Data Availability

All data underlying the results are available as part of the article and no additional source data are required.
