# Supplementary material for: Case Report: Tuberculous abscess of the popliteal fossa: A case report
Source: F1000Res. 2023 Sep 25;12:1194. [Version 1] doi: 10.12688/f1000research.138152.1 (PMC11079585; doi:10.12688/f1000research.138152.1)
Supplement: Peer review history

# Reviewer report: Reviewer response for version 1

**DOI:** 10.5256/f1000research.151331.r209226

**Published:** 2024-05-14

**Authors:** Mohamed Ben Jemaa

**Affiliations:** 1 Orthopedic Surgery Department - Habib Bourguiba Universty Hospital of Sfax, University of Sfax, Sfax, Tunisia

It presents a rare form of tuberculosis. This location remains rare also in endemic areas. This case report is well written and described. Clinical and paraclinical features are well analysed not only in the first paragraph of the clinical case but also in the paragraph of discussion. Figures provided by the authors are complete and englobe all clinical and paraclinical aspects. The conclusion is appropriate.

Are enough details provided of any physical examination and diagnostic tests, treatment given and outcomes?

Yes

Is the case presented with sufficient detail to be useful for other practitioners?

Yes

Is sufficient discussion included of the importance of the findings and their relevance to future understanding of disease processes, diagnosis or treatment?

Yes

Is the background of the case’s history and progression described in sufficient detail?

Yes

Reviewer Expertise:

orthopedic surgery, spine surgery, bone and joint infectious diseases, Extra-pulmonary Tuberculosis research Unit (University of sfax, faculty of medicine)

I confirm that I have read this submission and believe that I have an appropriate level of expertise to confirm that it is of an acceptable scientific standard.

# Reviewer report: Reviewer response for version 1

**DOI:** 10.5256/f1000research.151331.r271423

**Published:** 2024-05-08

**Authors:** Mouadh Nefiss

**Affiliations:** 1 Department of Orthopaedic Surgery, Mongi Slim University Hospital, Tunis, Tunisia

Dear author

 Thank you for the interesting case you submitted.

 the paper is well written, however I propose to rephrase certain sentences in particular:

 "On examination, the patient had a limp due to an antalgic stance in the knee flessum. An inflammatory swelling of 10 cm in diameter was found in the right popliteal fossa. It was a painful, soft, and mobile swelling"

 you can say: "the patient had a limp when walking due to knee flessum as well as a painful, inflammatory swelling of the popliteal fossa that was soft and mobile."

 I propose also to change: "The decision was to start an antituberculosis trial treatment......saphenous vein" with: "The treatment was based on anti-tuberculosis chemotherapy after pre-therapeutic assessment and for a total duration of 9 months. Anticoagulant treatment was prescribed for associated thrombophlebitis."

Are enough details provided of any physical examination and diagnostic tests, treatment given and outcomes?

Yes

Is the case presented with sufficient detail to be useful for other practitioners?

Yes

Is sufficient discussion included of the importance of the findings and their relevance to future understanding of disease processes, diagnosis or treatment?

Yes

Is the background of the case’s history and progression described in sufficient detail?

Yes

Reviewer Expertise:

spine surgery, orthopedic surgery

I confirm that I have read this submission and believe that I have an appropriate level of expertise to confirm that it is of an acceptable scientific standard.

# Reviewer report: Reviewer response for version 1

**DOI:** 10.5256/f1000research.151331.r213649

**Published:** 2024-02-08

**Authors:** Saoussen Miladi

**Affiliations:** 1 Faculty of Medicine of Tunis, Mongi Slim Hospital, Department of Rheumatolgy, University of Tunis El Manar, Tunis, Tunisia

- The authors report a case report of a tuberculous abcess of the popliteal fossa occurring on a 17 year old boy.
- This is an interesting case since the localisation of tuberculosis is rare in the popliteal fossa.
- The diagnosis relayed on anatomopathology typical findings.
- The case is well described and follow-up is mentioned.
- Figures are with good quality. 

Are enough details provided of any physical examination and diagnostic tests, treatment given and outcomes?

Yes

Is the case presented with sufficient detail to be useful for other practitioners?

Yes

Is sufficient discussion included of the importance of the findings and their relevance to future understanding of disease processes, diagnosis or treatment?

Yes

Is the background of the case’s history and progression described in sufficient detail?

Yes

Reviewer Expertise:

rheumatology, orthopedics

I confirm that I have read this submission and believe that I have an appropriate level of expertise to confirm that it is of an acceptable scientific standard.
